# Patient priority setting in HIV ageing research: exploring the feasibility of community engagement and involvement in Tanzania

**DOI:** 10.1186/s40900-022-00409-y

**Published:** 2023-02-17

**Authors:** Ellisiv Clarke, Grace Anderson-Saria, Aloyce Kisoli, Sarah Urasa, Susan Moloney, Ssenku Safic, Jane Rogathi, Richard Walker, Louise Robinson, Stella-Maria Paddick

**Affiliations:** 1 Newcastle University, Campus for Ageing and Vitality, Westgate Road, Newcastle Upon Tyne, NE4 6BE UK; 2 Anderson Memorial Rehabilitation and Care Organisation (AMRCO), Moshi, Tanzania; 3 Kilimanjaro Christian Medical University College, Moshi, Tanzania; 4 Mount Meru Hospital, Arusha, Tanzania; 5 Northumbria Healthcare NHS Foundation Trust, North Tyneside General Hospital, North Shields, UK; 6 Gateshead Health NHS Foundation Trust, Gateshead, UK

**Keywords:** HIV, HIV-associated neurocognitive disorders (HAND), Older adults, Sub-Saharan Africa, Community engagement and involvement (CEI), Patient and public involvement (PPI), Research priority setting, Research prioritisation

## Abstract

**Objective:**

The chronic complications of ageing with HIV are not well studied in sub-Saharan Africa (SSA) where general healthcare resources are limited. We aimed to collaborate with individuals living with HIV aged ≥ 50 years, and community elders (aged ≥ 60 years) living with non-communicable diseases in the Kilimanjaro region of Tanzania in a health research priority-setting exercise.

**Methods:**

We conducted structured workshops based on broad questions to aid discussion and group-based patient priority setting, alongside discussion of the feasibility of future community research engagement. Participant priorities were tallied and ranked to arrive at core priorities from consensus discussion.

**Results:**

Thirty older people living with HIV and 30 community elders attended separate priority setting workshops. Both groups reported motivation to participate in, conduct, and oversee future studies. In this resource-limited setting, basic needs such as healthcare access were prioritised much higher than specific HIV-complications or chronic disease. Stigma and social isolation were highly prioritised in those living with HIV.

**Conclusions:**

Community engagement and involvement in HIV and ageing research appears feasible in Tanzania. Ageing and non-communicable disease research should consider the wider context, and lack of basic needs in low-income settings. A greater impact may be achieved with community involvement.

**Supplementary Information:**

The online version contains supplementary material available at 10.1186/s40900-022-00409-y.

## Plain English summary

The population in sub-Saharan Africa is ageing. The majority of people living with HIV infection also live in Africa, and they are ageing now that treatment is widely available. Current research on the chronic complications of ageing with and without HIV in sub-Saharan Africa is very limited, meaning that little is known on how to improve symptoms. In this pilot study, researchers from Tanzania and the UK worked with older people living with HIV, and community elders in Tanzania in a health research priority-setting exercise. Thirty older people living with HIV and thirty community elders attended workshops where they listed issues important to them, and then voted for each item as a group. Priorities were ranked in order of importance by adding up the number of votes. We also asked how interested and motivated older people were to work jointly with academic researchers and what might help and support them to do this. Both groups reported that they felt very motivated to participate in, conduct and oversee future research studies. A key finding was that basic needs, such as being able to see a doctor regularly and buying medications, were prioritised much higher than specific HIV-complications or chronic disease. Stigma and social isolation were important issues for older people with HIV. Our pilot findings suggest that meeting basic needs should be a key part of future work on living and ageing with chronic disease in Tanzania. The importance of working with patients and communities is also highlighted.

**Supplementary Information:**

The online version contains supplementary material available at 10.1186/s40900-022-00409-y.

## Background

In the last decade, the successful expansion of combination anti-retroviral therapy (cART) in sub-Saharan Africa (SSA) has markedly increased life expectancy in people living with HIV [1]. The number of people with HIV aged 50 and over is projected to triple from 3.1 million in 2011 to 9.1 million by 2040 [2]. Despite a paucity of epidemiological studies focussing on the longer-term effects of HIV in the ageing population, this older age group are therefore predicted to become 1 in 4 of the total affected [3] and experience increasingly recognised chronic complications [4–6]. Optimised implementation of ART in Tanzania has the potential to reduce AIDS-related deaths [7] and transmission [8] but lead to ageing of the population with HIV as detailed above [9, 10].

Advocacy and patient-led initiatives have notably changed policy and increased cART HIV treatment access worldwide including amongst marginalized communities in SSA [11, 12]. Strong community engagement and involvement (CEI) are now core expectations of HIV interventional studies [13]. The objective of authentic community engagement and involvement (CEI) is to cultivate shared ownership and trust. When effective CEI results in locally-relevant findings resulting from mutual goal setting [14, 15]. To date, the focus of large-scale patient-led HIV advocacy initiatives has been on testing, monitoring and treatment to reduce transmission [16, 17] rather than ageing with chronic complications. Similarly, SSA health services, resources and data currently focus on infectious and acute illness rather than chronic disease and the different and complex health needs associated with ageing [18, 19]. None of these governmental or advocacy initiatives currently focus on the needs of this new population of older people living with HIV. Similarly, research data originate almost exclusively in high-income countries, and are unlikely to be transferable since people living with HIV in Africa differ socio-culturally, demographically, and in healthcare access, comorbidities and risk factor exposure. A wider complicating issue is that most collaborations are led by ‘Global North’ researchers and understanding of the perspectives and priorities of individuals in the Global South may be limited [20]. Newer participatory approaches such as community mapping aim to address this [21].

Similar to CEI, patient and public involvement (PPI) refers to work carried out ‘with’ or ‘by’ patients and community members rather than 'about ' or 'for' them, and recognises that patient and public perspectives may differ from those of researchers [22].

In international collaborations involving low and middle-income country (LMIC) populations and high-income country (HIC) researchers, three key theoretical imperatives of PPI/CEI are considered crucial, and are relevant to the context of this work. These are the emancipatory imperative, aiming to address power imbalances between vulnerable populations and researchers; the efficiency imperative, aiming to focus scarce research resources on questions prioritised by the population; and the political imperative, aiming to co-create knowledge between researchers and lay stakeholders [23, 24].

HIV-associated neurocognitive disorders (HAND) are a major chronic complication affecting an estimated 42.6% (16.1 million) of people with HIV worldwide [5], of whom the majority (72%) live in SSA [5]. Since most people living with HIV reside in SSA [1], HAND are likely to become a leading cause of cognitive impairment in Africa [5, 6], a burden not shared by high-income countries. There are 1.7 million people living with HIV in Tanzania, with adult prevalence estimated at 4.8% [25].

We recently published clinical data from the Kilimanjaro region of Tanzania indicating a high prevalence of HAND (47%, with 25% symptomatic) and DSM-IV major depression (16.6%) in people living with HIV aged 50 and over [26]. Wider SSA data remain extremely limited for this newly ageing population, and valid disease measures are also lacking [6, 27], potentially excluding African populations from future collaborative interventional and preventive studies.

Both older people, and those living with HIV, are potentially marginalised groups, impacted further by finite healthcare resources focussed on acute illness [28, 29]. We wished to understand the priorities of people living with HIV within the context of the wider ‘older’ population Whilst outcomes of our existing research collaboration in northern Tanzania (epidemiological studies of ageing and non-communicable disease) are regularly disseminated to a range of community, medical and government stakeholders through an established mechanism, we are not aware of any previously established ageing research CEI or PPI groups within Kilimanjaro that involve those affected by HIV. The feasibility of the approach within this context is therefore unknown. Good HIV participatory practice guidelines define community as ‘separate and overlapping groups of people affected by HIV in various ways’ [13]. Inclusion of people living with HIV alongside community members in which studies are embedded is good participatory practice.

## Aims

We aimed to collaborate with individuals living with HIV aged ≥ 50 years, and community elders (aged ≥ 60 years) living with non-communicable diseases but without diagnosed HIV in a health research priority setting exercise. We also aimed to explore the feasibility of using a CEI/PPI approach in future studies in Kilimanjaro.

## Methods

### Study design

This study took a workshop approach, considered effective in obtaining culturally specific information about values, opinions, and social contexts [30]. Due to resource and infrastructure challenges, use of an established priority-setting framework was not feasible. However we used elements of the James Lind Alliance (JLA) [31] framework to develop a prioritisation and ranking exercise for this study, considering both the ‘emancipation’ and ‘efficiency’ PPI imperatives relevant to vulnerable groups and low-resource settings (for detailed discussion guide, see Additional file 1). The principles of the JLA that we utilised were inclusivity, transparency and a commitment to using and contributing to the evidence base. We felt we did not meet the criteria for equal involvement as we recruited participants based on their previous knowledge and involvement with our research.

To support the integrity of our findings and the transparency of our decision making process, we used both the REPRISE [32] reporting framework and the GRIPP2 checklist [33] (for summaries, see Additional file 2).

The workshops focussed on four broad key questions,

1. What are the areas of ageing research most important in this community?
2. What potential interventions are most valued in this population?
3. How should future research be organised to better facilitate community participation?
4. How much involvement would this community like to have in the planning, conduct and dissemination of future research findings?

### Setting and participants

Two workshops were conducted in the Kilimanjaro region of Tanzania. Older people (aged ≥ 50 years) living with HIV were recruited from the HIV Care and Treatment clinics of Mawenzi Regional Referral Hospital (a government hospital) and St Joseph’s District Designated Catholic Hospital (charity funded) in Moshi urban district. Word of mouth recruitment, with convenience sampling was conducted by volunteer clinic coordinators who were also local patient representatives. Three clinicians from Mawenzi hospital attended the workshops as observers, supported by hospital management.

Individuals aged ≥ 60 years living with chronic disease were recruited from Mwika village in Kilimanjaro. Mwika residents were invited through the Anderson Memorial Rehabilitation and Care Organisation, a locally run Government-registered grassroots organisation aiming to improve healthcare access for community elders.

A lower age cut-off was used in HIV given that premature and/or accelerated ageing and increased chronic disease prevalence is hypothesised to occur in HIV [34]. These two age cut-offs were also representative of those used in previous local HIV and chronic disease studies [26].

### Ethical considerations

The Kilimanjaro Christian Medical University College research ethics committee and Tanzanian National Institute for Medical Research approved the study. CEI and PPI are often not considered to be research and therefore exempt from ethical review in high-income settings, but these approaches are relatively novel in Tanzania, and advice from senior clinicians was to obtain ethical review, due to inclusion of potentially stigmatised or vulnerable participants. Municipal and community leaders, and hospital managers, gave permission to conduct the workshops. Written informed consent to participate, and record group-level and individual anonymised responses was obtained from individual participants, after making a general announcement to clinic attendees about the aims and process of the workshops. Identifiable information was not shared, and personal details were only required to issue expenses receipts. Participants were reminded that HIV workshop attendance would indirectly disclose their status to other attendees. The importance of confidentiality was made clear individually and within the workshop ground rules. Travel expenses were paid and refreshments and meals provided to facilitate attendance, in line with NIHR community engagement guidelines [35].

HIV-related stigma is well recognised in Tanzania [36]. Our previous clinical experience suggested that many patients preferred to travel long distances to HIV services outside their locality, and discarded medicine boxes before leaving to avoid indirect disclosure of HIV status. Given the stigma, we organised separate workshops for people with HIV and other community residents.

### Workshop structure and organisation

Facilitators were local clinicians, nurses and occupational therapists selected based on experience in workshop facilitation, and of consent and data collection for ageing and HIV research studies. A workshop format was selected for feasibility, given resource constraints. Workshop participants were organised into small discussion groups each with a mix of demographics (sex, educational /occupational background). To facilitate free discussion the small groups were supported by a facilitator with similar local sociocultural experience. Ground rules relating to confidentiality and respecting the contribution of all workshop participants were established. Participants were encouraged to raise questions or concerns, before and during discussions. Each small group elected a chairperson to lead the discussion, a secretary to document priorities generated using flip charts and pens, and a timekeeper. Discussions were not video or voice-recorded, due to concerns these might limit free participation. All discussions took place in Swahili to promote inclusivity.

Facilitators opened discussion by first outlining the purpose of the workshop and the importance of understanding the priorities of the local community in the context of limited resources. Differences in healthcare, community and geography between high-income countries (where most previous studies had been conducted) and Tanzania were highlighted in order to promote discussion of the relevance to the Tanzanian context. Current knowledge gaps (from the perspective of the researchers) were further outlined by presenting the current limited data on ageing and high prevalence of non-communicable disease in Kilimanjaro, and the high prevalence of neurocognitive complications and mental health disorders in older people with HIV. Sharing of this information by facilitators who were also local clinicians was hoped to foster a sense of shared ownership of the results amongst workshop participants.

Priorities generated by each small group were clarified through whole group discussion (HIV and community elders separately) and then combined into a single list. Participants then voted (by raising hands) on each proposed priority to identify which they felt to be the most important. Common themes were tallied and prioritised according to frequency with the ten priorities ranked highest considered to be the group’s consensus priorities.

## Results

In total, 60 participants attended the workshops with three facilitators. Participants in Moshi (HIV clinics, n = 30) ranged in age from 51 to 81 (median 68) and 18/30 were female. Participants in Mwika (community, n = 30) ranged in age from 61 to 90 (median 76) and 19/30 were female.

The most common themes arising from each of the core questions explored during the workshops are presented in Table 1: Ageing research priorities, Table 2: Views on community participation in future health research, and Table 3: Aspects of health research participants would be willing to participate in. Rankings given by older people living with non-communicable disease and people living with HIV are listed separately in each table. A full list of generated concerns, and associated tallies is presented in the Additional file 3.

**Table 1:** Responses to key questions 1 and 2. Ageing research priorities

Order of priorities	Community elders workshop	HIV clinic workshop
*Key question 1: What ageing research priorities could make the biggest impact in this community?*		
1	Basic needs including food, shelter and clothes	Income of the elderly
2	Nutritional status of the elderly	Geriatric disease research
3	Provision of health services to the elderly	Nutritional status of the elderly
4	Income of the elderly	Basic needs including food, shelter and clothes
5	Addressing non-communicable diseases	Provision of health services to the elderly
6	Geriatric disease research	Social interaction
7	Urinary tract disorders	Stigmatisation of the elderly and ageing
8	Home care services	Non-communicable diseases
9	Living conditions	Urinary tract disorders
10	Frailty	Neurological disorders and dementia

Order of priorities	Community elders	HIV clinic workshop
*Key question 2: What interventions are most valued in this population?*		
1	Public education on health of the elderly	Health insurance
2	Public education regarding modifiable risk factors	Free geriatric health services
3	Health insurance	Provision of a balanced diet
4	Free geriatric health services	Old people to be loved, regularly visited and respected
5	Improvement of living environment for elderly	Transport to healthcare services
6	Effective implementation of health polices	Government attention and involvement in the needs of the elderly
7	Education on nutrition	Effective implementation of health polices
8	Government grants to support elderly people in agriculture	Government grants to support elderly people in agriculture
9	Provision of a balanced diet	Regular health check-ups
10	Establishment of nursing homes	Commemoration day for the elderly in rural areas

**Table 2:** Views on community participation in future health research

Priorities	Community elders	HIV clinic workshop
*Key Question 3: How can research be organised to better facilitate and encourage community participation?*		
1	Education regarding research	Education regarding research
2	Good communication among community members and researchers	Simple and understandable language to be used
3	Communication through existing community structures	The benefit and importance of the research to be clearly explained
4	Community meeting before the research	Updates on previous research
5	Updates on previous research	Involvement in planning stages
6	Community and government organisation involvement	Appointment of community leaders to inform and educate the community on research
7	Motivation to be given to the elderly e.g. time compensation	Privacy and confidentiality must be maintained
8	Simple and understandable language to be used	Clear documented consent
9	Transparency regarding ethics etc	Good communication among community members and researchers
10	The benefit and importance of the research to be clearly explained	Communication through existing community structures

Priorities	Community elders	HIV clinic workshop
*Key Question 4: How much involvement would the community like to have in the planning, conduct and dissemination of research findings?*		
1	Timely dissemination of findings and associated explanation/ counselling	Involvement in planning stages
2	Involvement in planning stages	Community attendance and acceptance of findings at dissemination of results
3	Informing the community of plans to conduct research early on	Community cooperation as participants
4	Community cooperation as participants	Timely dissemination of findings and associated explanation/ counselling
5	Community acceptance of intervention	Education in importance of giving proper and true research answers
6		Involvement in implementation of interventions
7		Conduct of research

**Table 3:** Aspects of health research in which participants reported willingness to participate

	Community elders	HIV clinic workshop
Planning the study and selecting assessment tools and measures	9	11
Being trained to carry out the study (data collection)	30	30
Reviewing documents (patient information sheet, ethics documents etc.)	4	4
Disseminate study findings	4	6
Provide overall oversight on conduct and research priorities	30	30

### Ageing research priorities (Table 1)

The major priorities in both groups centred on basic needs, including nutrition, shelter, clothing and income, above specific health needs. Both groups valued general health provision for older people. Participants of the HIV disease workshop ranked geriatric (older persons) disease research higher than the community participants did. Other differences were that participants of the HIV workshop prioritised social interaction and removal of stigma, issues not raised by the community participants who instead prioritised home care services and frailty.

Both groups prioritised nutritional support, and government support of older people working in subsistence farming and agriculture. Similarly, both groups prioritised increased healthcare access and cost reductions in health services. Community elders suggested and prioritised public health education and risk factor reduction, but participants of the HIV group did not.

### Views on community participation in future health research (Table 2)

Both groups prioritised high-quality communication from the initial planning stages until the dissemination of results in future research for encouraging community participation. Educating the local community, integration with existing community structures and clarity of language were also highly prioritised by both groups. Suggestions for information exchange included involvement of religious congregations, schools and local media alongside community stakeholder meetings in planning new studies. Frequent and high-quality communication between research teams and community members was considered essential. Participants of the HIV workshop prioritised maintaining confidentiality of research participants and clear, documented consent, but the community elders group did not. In terms of involvement preferences, participants from both workshops were keen for involvement in all stages of research. Suggestions were made for small groups of study participants to continue to meet and identify community research priorities.

#### Aspects of health research participants would be willing to participate in (Table 3)

All respondents said they would be willing to be trained in data collection for future studies and to provide oversight on research conduct. There was less enthusiasm for selection of assessment tools, reviewing documents such as patient information sheets, and dissemination of study findings. Reasons for these preferences were not stated. As well as reiterating the importance of informed consent in research, respondents suggested education regarding the benefits of research would help foster trust in the process and enable high-quality engagement and involvement by community members.

## Discussion

### Research priority setting

Several overarching priorities with a strong public health focus were identified by both groups. Although cancer, specific infectious diseases and chronic conditions including dementia were all mentioned as priorities, major priorities were governmental and community support to meet basic needs. Adequate nutrition and income, general healthcare needs, structural and social aspects of healthcare, and social stigma were identified as key issues.

It is perhaps not surprising that our workshop participants prioritised basic needs and healthcare access. A 2011 Tanzanian report indicated that 45% of older people rely on family for income, with 28% in full-time work, 7% in part-time work and others relying on remittances (21%), pensions (5%) and charitable donations, Non-Governmental Organisations (NGOs) and neighbours (2%) [37]. Almost 82% of Tanzanian elders aged 60 and older live rurally, which increases vulnerability to financial, food and housing insecurity [37]. Frailty is associated with food insecurity [38], and estimated local prevalence of frailty in Kilimanjaro is 11% [39]. Food insecurity is associated with functional impairment [40] and higher chronic disease prevalence [41].

Healthcare in Tanzania is part-subsidised by government but supplemented by out-of-pocket payments at a cost-sharing (not cost-recovery) rate [42]. As age and comorbidities increase, increasing costs of healthcare visits alongside decreasing physical ability to work may result in financial barriers to accessing health services. This could also explain prioritisation of basic and healthcare needs.

Although groups raised common themes, there were some differences between the priorities of people living with HIV and those of community elders. Healthcare access appeared a greater concern for community elders than for people living with HIV. Our previous research suggests that individuals on treatment for HIV may have better-managed and better recognised chronic comorbidities through access to regular clinic review funded through international HIV aid programmes which may not be similarly available to the general population [43].

Public and community education for older people was also prioritised. Suggestions included smoking cessation or alcohol reduction advice, as well as carers’ education programmes for families caring for frail elders. Again, the rural elders more commonly prioritised these. This may reflect increasing local awareness of alcohol and tobacco smoking as risk factors for non-communicable disease [44, 45]. It may also be that widespread HIV-focussed public education programs, and healthcare advice available to people under regular HIV follow up, mean that this health education is less of a perceived need for this group.

People living with HIV prioritised social isolation, stigma and the importance for older people to be loved and respected. Strong links exist between frailty and social isolation or loneliness in high income countries [46], although SSA data are lacking. However, available local data on ageing with HIV suggests that both frailty and HIV-associated neurocognitive disorders (HAND) may be associated with social isolation [47, 48].

### Feasibility of PPI/CEI in HIV ageing research

Facilitators of the workshops reported excellent participant engagement, with open dialogue and discussion, providing anecdotal evidence that further community and patient involvement in HIV and ageing research would be feasible in this setting. The high level of interest in future involvement in the planning and conduct of research indicated amongst respondents is also highly encouraging.

Despite prominence in national development priorities outlined by international literature, CEI and PPI are not yet widely implemented in SSA [49]. One barrier to CEI in HIV-infected populations might be the ongoing stigma surrounding HIV infection in Tanzania [36]. Fear of HIV status becoming public knowledge may result in reluctance to participate in studies. Concomitantly, older interventional studies with poor community engagement strategies, such as those of pre-exposure prophylaxis, have seriously eroded trust between people with HIV and researchers [50].

Whilst participants in our workshops were generally eager to engage with research, those living with HIV were especially keen to be involved with planning and priority setting. This could be a reflection of the design and delivery of the study workshops, which deliberately respected participant confidentiality and promoted ownership and equality of voice. Participants from the HIV clinic might also have gained confidence in and familiarity with the concept of medical research, due to exposure to ongoing clinical studies taking place in the clinics they attend.

It has been suggested that community engagement in global health contexts has undergone a ‘reflexive shift’ with increasing recognition that much community engagement assumes a knowledge gap to be addressed in a ‘top down’ manner by researchers informing the public, rather than a truly collaborative process [51]. It was interesting therefore, that provision of research education and information by researchers to the local community were repeatedly prioritised, especially by community elders. Our research team have a close historical relationship with this community, having engaged them in research over a period of the last several years and disseminated findings on an annual basis. This familiarity with both with our team and our research itself may explain the high regard in which our rural participants prioritise dissemination of study results. The outcome of this workshop will also be disseminated to the wider community, stakeholders and district medical office at these annual update meetings.

Consideration of the key purpose of community engagement in global health research is important. Goals may include increasing recruitment and retention, by accommodating local sociocultural factors and increasing community ‘buy in’. Whilst this may be a useful goal, it is researcher driven. We wished to understand research priorities from the perspective of people living with HIV, necessitating a more collaborative approach. The workshop priorities generated indicate that rather than simply being ‘educated’ and ‘informed’ about the benefits of research on cognitive impairment, participants clearly indicated that broader aspects of health and social care were equally, if not more, important. This study demonstrates that without working to co-produce research questions, we may fail to address the issues of most importance to the groups we are intending to benefit.

Patient-led campaigns, advocacy, and participation in HIV research have been highly successful in some contexts [11, 12, 15, 16]. However, such efforts have typically addressed treatment access and prevention (the focus of most advocacy initiatives to date), rather than management of chronic complications and ageing. This newly ageing population might not yet have a voice in the same way. Our workshop participants did not prioritise treatment access or prevention, possibly because these issues are now successfully addressed.

## Strengths and limitations

A strength of this work is that it is underpinned by JLA principles and the workshop design included elements of the JLA process. A further strength is that review against the REPRISE framework indicates that many REPRISE domains are addressed by the study design and delivery. However, when considering the theoretical underpinnings of PPI, although we obtained some insights related to the ‘efficiency’ imperative and local priorities, the ‘emancipation’ imperative was more challenging as questions and context were framed by facilitators with guidance from study co-investigators. The lack of a previously established PPI group, and exploratory nature of this work contributed to this issue, which may be addressed in future initiatives. The literature around PPI/CEI in the LMIC setting is very limited. What is available suggests that, as in our work, studies have focussed almost exclusively on research planning, rather than evaluation and impact, and that findings are primarily reported from the perspectives of the researchers [52]. Future work should aim to address these issues by collaborating with stakeholders throughout projects to include evaluation and reporting by stakeholders rather than researchers [53].

Although this study suggests CEI/PPI is feasible in HIV ageing research in this setting, a common challenge is that those already ‘engaged’ are more likely to participate [51]. Our community participants had previously engaged with a grassroots health organisation, and HIV clinic participants were regularly attending clinic follow-up. With regard to vulnerable populations and power imbalances, challenges related to comorbidities, access and transport have the potential to further accentuate inequalities and the gap between included and excluded [51]. Specifically in relation to our study, though we supported transport for CEI activities, our pool of HIV clinic participants were those able to regularly access transport to a clinic, and with the physical and psychosocial resources to manage regular clinic attendance and medication collection. Similarly, recruitment of community elders previously engaging with community groups related to health might bias viewpoints towards more motivated individuals, with personal and social resources to allow participation in these activities. The identified priorities may not reflect those of more stigmatised, marginalised and seldom heard groups who are not actively engaged in HIV clinic follow-up or those community elders not actively involved in community groups.

Non-heterosexual sexual activity is criminalised in Tanzania [54], and clinics servicing ‘key populations’ e.g. men who have sex with men (MSM) have been closed by the Ministry of Health due to concerns about promoting unethical behaviour [55]. Therefore, there are likely to be unheard voices from this community that were not captured in our workshops, for these reasons. This is important as marginalised communities disproportionately contribute to HIV transmission in SSA despite being an overall minority [56]. This pilot work does not address the emancipation imperative for these groups, and future PPI and CEI work should consider other, anonymised ways of contributing without workshop attendance.

Women were overrepresented in the workshops, comprising nearly double the number of male participants. HIV is more prevalent in women than men in Tanzania [57]. Notwithstanding the marginalisation of MSM, future priority setting exercises should aim to include a more balanced split of men and women to maximise relevance.

Our participants with HIV were from one, urban site (although some travelled from rural areas) whereas community elders all lived rurally. This allowed for confidentiality of participants regarding HIV status, and may have encouraged participation. However, to contextualise the differences between the two groups, it would have been useful to include rural residents with HIV and urban community elders. This was unfortunately not possible in this current study due to resource limitations.

## Conclusion

This novel study based in Tanzania, suggests that a CEI/PPI approach to priority setting is feasible, and has the potential to benefit both researchers and community members.

We found that there was motivation amongst both community elders and older people living with HIV to participate in, conduct, and oversee future studies. Clear communication between researchers and the community was an overarching theme in these workshops.

In this resource-limited setting, basic needs such as healthcare access were prioritised over the specific complications of HIV or chronic disease explored in recent local epidemiological studies. Future non-communicable disease research should consider the wider context and the importance of basic needs. Although biomedical research (in our case, the neuropsychiatric complication of ageing with HIV, and diagnosis and management of dementia in Africa) has its value, benefit is limited if patients cannot access, or afford to pay for, healthcare. Future research should include community involvement and engagement at all stages, in line with these findings.

## Supplementary Information

**Additional file 1:** Individual participant responses and tallies to each main question.

**Additional file 2:** GRIPP 2 Checklist Summary.

**Additional file 3:** REporting guideline for PRIority SEtting of health research (REPRISE) domains and sub-items summary

## Data Availability

All data generated or analysed during this study are included in this published article [and its supplementary information files] other than deidentified demographic data that are available from the authors upon reasonable request.
