# Supplementary material for: Patient priority setting in HIV ageing research: exploring the feasibility of community engagement and involvement in Tanzania
Source: Res Involv Engagem. 2023 Feb 17;9:3. doi: 10.1186/s40900-022-00409-y (PMC9938604; doi:10.1186/s40900-022-00409-y)
Supplement: Supplementary file 1 — Additional file 1: Individual participant responses and tallies to each main question. [file 40900_2022_409_MOESM1_ESM.docx]

**Supplementary data– Individual participant responses and tallies to each main question**

| **What aspects of ageing research will make the biggest impact to PLWH and their communities?** | **PLWNCD** | **PLWH** |
| --- | --- | --- |
| Basic needs including food, shelter and clothes | 5 | 3 |
| Income of the elderly | 4 | 5 |
| Nutritional status of the elderly | 5 | 4 |
| Provision of health services to the elderly | 5 | 2 |
| Home care services | 2 | 0 |
| Geriatric disease research | 3 | 5 |
| Non-communicable diseases | 4 | 1 |
| Mirathi (inheritance) | 1 | 0 |
| Living conditions | 2 | 1 |
| Communicable diseases | 1 | 0 |
| Home visits | 1 | 0 |
| Neurological disorders including dementia | 1 | 1 |
| Blood disorders | 1 | 0 |
| Frailty | 2 | 0 |
| Respiratory infections | 2 | 0 |
| Urinary tract disorders | 3 | 1 |
| Social stressors including childcare | 2 | 0 |
| Value of the elderly in the society | 1 | 0 |
| Behavioural changes | 1 | 0 |
| Social interaction | 2 | 2 |
| Transport to healthcare services | 1 | 0 |
| Public education on elderly care | 1 | 0 |
| Nursing care and residential homes | 1 | 1 |
| Eye diseases | 1 | 1 |
| Cancer | 1 | 1 |
| Stigmatisation of the elderly and ageing | 0 | 2 |
| **What interventional models are most likely to be acceptable in this population?** | **PLWNCD** | **PLWH** |
| Health insurance | 2 | 3 |
| Free geriatric health services | 2 | 2 |
| Public education on health of the elderly | 4 | 0 |
| Improvement of living environment for elderly | 2 | 0 |
| Effective implementation of health polices | 2 | 1 |
| Education on nutrition | 2 | 0 |
| Public education regarding modifiable risk factors | 3 | 0 |
| Government grants to support elderly people in agriculture | 2 | 1 |
| Establishment of nursing homes | 1 | 0 |
| Provision of a balanced diet | 2 | 2 |
| Establishment of geriatric department to the hospitals | 1 | 0 |
| Old people to be loved, regularly visited and respected by their family members and community at large | 1 | 2 |
| Geriatrics-focussed research | 1 | 0 |
| Community participation in research | 1 | 0 |
| Socioeconomic support for the elderly | 1 | 0 |
| Greater community involvement of the elderly | 1 | 0 |
| Elderly people to be afforded leadership positions | 1 | 0 |
| Regular health check-ups | 1 | 1 |
| Involvement and priority in community activities | 1 | 0 |
| Transport to healthcare services | 1 | 2 |
| Government attention and involvement in the needs of the elderly | 0 | 2 |
| Commemoration day for the elderly in rural areas | 0 | 1 |
| Government pension | 0 | 1 |
| **How can this research be organised to better facilitate community participation?** | **PLWNCD** | **PLWH** |
| Education regarding research | 9 | 4 |
| Community and government organisation involvement | 1 | 0 |
| Motivation to be given to the elderly e.g. time compensation | 1 | 0 |
| Simple and understandable language to be used | 1 | 4 |
| Good communication among community members and researchers | 3 | 1 |
| Communication through existing community structures | 3 | 1 |
| Community meeting before the research | 3 | 0 |
| Transparency regarding ethics etc. | 1 | 0 |
| The benefit and importance of the research to be clearly explained | 1 | 4 |
| Updates on previous research | 3 | 2 |
| Regular research to be conducted to help retain and build on knowledge | 1 | 0 |
| Involvement in planning stages | 0 | 2 |
| Education regarding community rights to involvement in research | 0 | 1 |
| Appointment of community leaders to inform and educate the community on research | 0 | 2 |
| Privacy and confidentiality must be maintained | 0 | 2 |
| Clear documented consent | 1 | 2 |
| Opinions are respected and integrated into research | 0 | 1 |
| **How much involvement would the community like to have in the planning, conduct and dissemination of research findings?** | **PLWNCD** | **PLWH** |
| Involvement in planning stages | 2 | 10 |
| Informing the community of plans to conduct research early on | 1 | 0 |
| Community cooperation as participants | 1 | 1 |
| Timely dissemination of findings and associated explanation/ counselling | 3 | 1 |
| Community acceptance of intervention | 1 | 0 |
| Community attendance and acceptance of findings at dissemination of results | 0 | 2 |
| Education in importance of giving proper and true research answers | 0 | 1 |
| Involvement in implementation of interventions | 0 | 1 |
| Conduction of research | 0 | 1 |
