# Supplementary material for: Patient priority setting in HIV ageing research: exploring the feasibility of community engagement and involvement in Tanzania
Source: Res Involv Engagem. 2023 Feb 17;9:3. doi: 10.1186/s40900-022-00409-y (PMC9938604; doi:10.1186/s40900-022-00409-y)
Supplement: Supplementary file 3 — Additional file 3: REporting guideline for PRIority SEtting of health research (REPRISE) domains and sub-items summary [file 40900_2022_409_MOESM3_ESM.docx]

The REporting guideline for PRIority SEtting of health research (REPRISE) domains and sub-items as related to our study

Key reference, and use of standardised format from Tong, A., Synnot, A., Crowe, S. et al. Reporting guideline for priority setting of health research (REPRISE).  BMC Med Res Methodol 19, 243 (2019). https://doi.org/10.1186/s12874-019-0889-3

| **No** | **Item** | **Descriptor and/or examples from REPRISE** | **Our study** |
| --- | --- | --- | --- |
| A | Context and scope |  |  |
| 1 | Define geographical scope | Global, regional, national, city, local area, institutional/organizational level, health service | The Kilimanjaro region of Tanzania, with potential extrapolation to other similar geographical settings in low-middle income African countries with high HIV prevalence. |
| 2 | Define health area, field, focus | Disease or condition specific, interventions, healthcare delivery, health system | Chronic complications (particularly neurocognitive) of living with treated HIV in Kilimanjaro. Since recent studies have established the scale of the issue locally, what should be the research priorities moving forward considering the limited available resources? |
| 3 | Define the intended beneficiaries | This may include the general population or a specific population based on demographic (age, gender), clinical (disease, condition), or other characteristics who may benefit from the research | Older people living with HIV attending urban Kilimanjaro clinics (aged 50 and over). Predefined contrasting comparison group, people living with chronic disease in rural Kilimanjaro (aged 60 and over) in contact with a local NGO for elders with chronic disease. |
| 4 | Define the target audience of the priorities | Policy makers, funders, researchers, industry or others who have the potential to implement the priorities identified | Researchers and research funders of HIV and wider Global Health and ageing work. |
| 5 | Identify the research area | Public health, health services research, clinical research, basic science | Clinical and public health research. |
| 6 | Identify the type of research questions | Etiology, diagnosis, prevention, treatment (interventions), prognosis, health services, psychosocial, behavioral and social science, economic evaluation, implementation; this may not be pre-defined | These were not pre-defined, but guided to include not only epidemiology (numbers affected). Priorities generated by participants focussed on aetiology, diagnosis prevention and treatment of specific conditions, but also wider public health context and healthcare access. |
| 7 | Define the time frame | Interim, short-term, long-term priorities, plans to revise and update | These were intended as interim priorities based on the situation of current very limited data. |
| B | Governance and team |  |  |
| 8 | Describe the selection and structure of the leadership and management team | Those responsible for initiating, developing, and guiding the process for priority setting, and examples of structures include; Steering Committee, Advisory Group, Technical Experts | The process was initiated and guided by core collaborators from Kilimanjaro Christian Medical University College and Newcastle University who had previously conducted epidemiological research on ageing with chronic disease in these communities. |
| 9 | Describe the characteristics of the team | Stakeholder group or role, institutional affiliations, country or region, demographics (e.g. age sex), discipline, experience, expertise | Leads were Dr Stella-Maria Paddick (old age psychiatrist with expertise in HIV and dementia research in Africa) Newcastle University, UK and Dr Sarah Urasa (neurologist and head of hospital services, with experience of HIV, ageing and dementia research in Africa) Kilimanjaro Christian Medical University College. |
| 10 | Describe any training or experience relevant to conducting priority setting | Consultants or advisors, members with experience or skills relevant to the conducting priority-setting e.g. qualitative methods, surveys, facilitation | Facilitators were chosen for proven previous experience of conducting dissemination workshops in diverse local communities, and facilitating workshops in other contexts as well as collecting qualitative data for research studies. They were also previously trained in informed consent. |
| C | Framework for priority setting |  |  |
| 11 | State the framework used (if any) | James Lind Alliance, COHRED, CHNRI, Dialogue Model, no framework (general research priority setting) | This was general research priority setting (no formal framework) but based on the principles of the James Lind Alliance |
| D | Stakeholders or participants |  |  |
| 12 | Define the inclusion criteria for stakeholders involved in priority-setting | Patients, caregivers, general community, health professionals, researchers, policy makers, non-governmental organizations, government, industry; specific groups including vulnerable and marginalized populations | HIV clinic stakeholders had to be established on HIV treatment attending one of two local clinics, and registered for routine follow-up from the clinic. They were 50 years or older as evidenced by their clinic card.  Community chronic disease stakeholders were resident in the Kilimanjaro area (in or near to Mwika village), aged 60 years and above and who had come into contact with the AMRCO (Anderson Memorial Rehabilitation and Care Organisation) a local NGO assisting with long term health and care needs. |
| 13 | State the strategy or method for identifying and engaging stakeholders | Partnership with organizations, social media, recruitment through hospitals | Participants were recruited by word of mouth, by patient coordinators (a volunteer, non-clinical role) at each HIV clinic who were able to discuss the workshops with patients as they arrived for routine appointments to collect medications etc. Volunteer coordinators are well known to patients and advise on practical issues, but are also not clinicians. Informed consent was subsequently obtained by one of the facilitators, trained in these procedures who subsequently spoke to individuals interested in taking part. |
| 14 | Indicate the number of participants and/or organizations involved | Number of individuals and organizations, include number by stakeholder group | These are detailed in the Results (description of the baseline sample recruited) |
| 15 | Describe the characteristics of stakeholders | Stakeholder group, demographic characteristics, areas of interest and expertise, discipline, affiliations | These are detailed in the Results (description of the baseline sample recruited) |
| 16 | State if reimbursement for participation was provided | Cash, vouchers, certificates, acknowledgement; what purpose e.g. travel, accommodation, honorarium | Refreshments and lunch were provided during the workshop and travel costs to and from the workshop were refunded. |
| E | Identification and collection of research priorities |  |  |
| 17 | Describe methods for collecting initial priorities | Methods e.g. Delphi survey, surveys, nominal group technique, interviews, focus groups, meetings, workshops; prioritization e.g. voting, ranking; mode e.g. face-to-face, online; may be informed by evidence e.g. systematic reviews, reviews of guidelines/other documents, health technology assessment | The core researchers designing the study were aware of limited systematic review data, and had personally collaborated on the existing epidemiological surveys recently published on the topic (ageing with treated HIV) in the local area.  Priorities were formulated based on the following questions designed as a framework for the workshops.  In older people living with HIV attending an urban Kilimanjaro clinic (aged 50 and over) and in people living with chronic disease in rural Kilimanjaro (aged 60 and over)   1. What are the areas of ageing research most important in this community? 2. What potential interventions are most valued in this population? 3. How should future research be organised to better facilitate community participation? 4. How much involvement would this community like to have in the planning, conduct and dissemination of future research findings? |
| 18 | Describe methods for collating and categorizing priorities | Taxonomy or other framework used to organize, summarise, and aggregate topics or questions | Tally and ranking of responses, with the 10 most frequently endorsed placed in a priority list, in a similar manner to some aspects of the James Lind Alliance framework. |
| 19 | Describe methods and reasons for modifying (removing, adding, reframing) priorities | Based on scope, clarity, definition, duplication, other criteria | All priorities raised were carefully discussed in order to understand the meaning, and if they were duplications of other priorities already raised.  The framing questions were used to identify any suggestions completely out of scope for example those related to pregnant women or children. Participants were reminded the focus was older persons health. |
| 20 | Describe methods for refining or translating priorities into research topics or questions | Reviewed by Steering Committee or project team | This was not completed as part of our study. Workshop participants agreed on research priorities, but these were not subsequently formulated into research questions by researchers as this was exploratory work |
| 21 | Describe methods for checking whether research questions or topics have been answered | Systematic reviews, evidence mapping, consultation with experts | Review of prior systematic reviews (including African data) and consultation with experts. |
| 22 | Describe number of research questions or topics | Number of priorities at each stage of the process | Stage 1 – unlimited.  Stage 2 – reduced to 10 per setting. |
| F | Prioritization of research topics/questions |  |  |
| 23 | Describe methods and criteria for prioritizing research topics or questions | Methods e.g. Delphi survey, surveys, nominal group technique, interviews, focus groups, meetings, workshops; Prioritization e.g. voting, ranking; Mode e.g. face-to-face, online; Criteria e.g. need, feasibility, novelty, equity | Method – workshops.  Prioritisation – voting and total of votes in second round.  Mode – face to face workshop using written record (flipcharts) and photographs of blackboard etc.  Criteria – greatest need and importance to particular participants. |
| 24 | State the method or threshold for excluding research topics/questions | Thresholds for ranking scores, proportions, votes; other criteria | Number of votes in second discussion was used to rank scores and determine the 10 most important research topics and questions. |
| G | Output |  |  |
| 25 | State the approach to formulating the research priorities | Area, topic, questions, PICO (population, intervention, comparator, outcome) | The current evidence remains extremely scant. Therefore topics explored were broad and priorities listed as broad areas for further exploration in future workshops. |
| H | Evaluation and feedback |  |  |
| 26 | Describe how the process of prioritization was evaluated | Survey, workshop | The exercise was conducted as a workshop with preparatory/planning activities |
| 27 | Describe how priorities were fed back to stakeholders and/or to the public; and how feedback (if received) was addressed and integrated | Public meetings or workshop, newsletters, website, email, online presentations | Priorities were fed back to participants at the end of the session. They were discussed within the research collaboration and written into a report (initially prepared by the facilitators) shared with the hospital managers and redrafted as a research paper by the core research collaborators. |
| I | Implementation |  |  |
| 28 | Outline the strategy or action plans for implementing priorities | Communication with target audience, via policies and funding | The strategy is to integrate community participation in future local studies as per the feasibility data and stated willingness of local groups to participate, and to collaborate on future research taking into account these stated priorities. |
| 29 | Describe plans, strategies, or suggestions to evaluate impact | Integration in decision-making, funding allocation, review of relevant documents | The impact will become clear if funded projects focussing on the priority areas is secured. The intention is to move forward to obtain such funding. |
| J | Funding and conflict of interest |  |  |
| 30 | State sources of funding | Name sources of funding for the priority-setting exercise; if relevant include the budget and/or cost | This work was part funded by the DePec study, but workshops and transport etc were funded by a Newcastle University Global Challenges Rapid Response grant (amount allocated for this Tanzanian work £1200) |
| 31 | Declare any conflicts or competing interests | State any conflicts of interest that may be at an individual level and/or at a contextual level (e.g. political issues, controversies) that may affect the process, output or implementation. | Important potential conflicts of interest include the previous focus of core research collaborators on cognitive impairment, dementia, neurology and geriatric medicine thereby potentially favouring these areas of research whether consciously or unconsciously (although we conducted these workshops to try to understand these potential differences in participant-led priorities).  Another major potential area of contextual conflict of interest is the fact that same sex relationships remain illegal in Tanzania, as does suicide and therefore it is likely participants felt unable to raise these issues, despite confidentiality safeguards. |
